# Dihydromyricetin enhances glucose uptake by inhibition of MEK/ERK pathway and consequent down‐regulation of phosphorylation of PPARγ in 3T3‐L1 cells

**DOI:** 10.1111/jcmm.13403

**Published:** 2017-11-21

**Authors:** Lei Liu, Min Zhou, Hedong Lang, Yong Zhou, Mantian Mi

**Affiliations:** ^1^ Research Center for Nutrition and Food Safety Chongqing Key Laboratory of Nutrition and Food Safety Institute of Military Preventive Medicine Third Military Medical University Chongqing China; ^2^ Department of Clinic Nutrition People's Hospital of Chongqing Banan District Chongqing China

**Keywords:** dihydromyricetin, type 2 diabetes, glucose uptake, insulin sensitivity, peroxisome proliferator‐activated receptor γ, mitogen‐activated protein kinase

## Abstract

Accumulating evidence suggests that inhibition of mitogen‐activated protein kinase signalling can reduce phosphorylation of peroxisome proliferator‐activated receptor γ (PPARγ) at serine 273, which mitigates obesity‐associated insulin resistance and might be a promising treatment for type 2 diabetes. Dihydromyricetin (DHM) is a flavonoid that has many beneficial pharmacological properties. In this study, mouse fibroblast 3T3‐L1 cells were used to investigate whether DHM alleviates insulin resistance by inhibiting PPARγ phosphorylation at serine 273 *via* the MEK/ERK pathway. 3T3‐L1 pre‐adipocytes were differentiated, and the effects of DHM on adipogenesis and glucose uptake in the resulting adipocytes were examined. DHM was found to dose dependently increase glucose uptake and decrease adipogenesis. Insulin resistance was then induced in adipocytes using dexamethasone, and DHM was shown to dose and time dependently promote glucose uptake in the dexamethasone‐treated adipocytes. DHM also inhibited phosphorylation of PPARγ and ERK. Inhibition of PPARγ activity with GW9662 potently blocked DHM‐induced glucose uptake and adiponectin secretion. Interestingly, DHM showed similar effects to PD98059, an inhibitor of the MEK/ERK pathway. DHM acted synergistically with PD98059 to improve glucose uptake and adiponectin secretion in dexamethasone‐treated adipocytes. In conclusion, our findings indicate that DHM improves glucose uptake in adipocytes by inhibiting ERK‐induced phosphorylation of PPARγ at serine 273.

## Introduction

The global prevalence of diabetes has risen from 4.7% in 1980 to 8.5% in 2014 1. Overweight and obesity, which are the strongest risk factors for type 2 diabetes, are estimated to cause a large proportion of the global diabetes burden 2. Unlike unmodifiable risk factors, such as genetics, ethnicity and age, overweight and obesity can be modified through behavioural changes and measures to combat obesity are crucial to halting the rise in diabetes. Besides storing energy, adipose tissue secretes many adipokines, which are involved in glucose and lipid metabolism. In the obese state, secretion of proinflammatory adipokines, such as tumour necrosis factor (TNF)‐α and interleukin (IL)‐6, is increased while secretion of insulin‐sensitizing adipokines, such as adiponectin, is decreased. This leads to an increase in macrophage infiltration and inflammatory response, as well as impairment of insulin signalling and glucose uptake 3, 4.

Peroxisome proliferator‐activated receptor γ (PPARγ) is a ligand‐activated transcription factor that modulates metabolism of glucose and lipid. PPARγ is predominantly expressed in adipose tissue and is essential for the differentiation and function of adipocytes 5. Previous studies have shown that cyclin‐dependent kinase 5 (CDK5)‐mediated phosphorylation of PPARγ at serine 273 (Ser273) increases expression of TNF‐α, IL‐1 and resistin, and decreases expression of adiponectin. This suggests that the anti‐diabetic mechanism of PPARγ ligands may involve inhibition of phosphorylation at Ser273 6. However, another key function of PPARγ is adipogenesis. Rosiglitazone (ROSI) and other members of the thiazolidinedione (TZD) class of anti‐diabetic drugs, which are full agonists of PPARγ, exert excellent insulin‐sensitizing effects, increase lipid accumulation, promote differentiation of fat cells and stimulate gene expression in fat cells 7. Interestingly, partial or non‐agonist PPARγ ligands, which either completely lack transcriptional activity or show weak activity compared with classical full agonists, nevertheless effectively block phosphorylation of PPARγ at Ser273 6, 7. A recent study found that both CDK5 and extracellular signal‐regulated kinase (ERK) directly phosphorylated PPARγ at Ser273 whilst CDK5 suppressed ERK through direct action with mitogen‐activated protein kinase (MEK) 8. These results suggest that regulation of the ERK/CDK5 axis might hold promise for the treatment of type 2 diabetes.

In southern China, *Ampelopsis grossedentata* is used to make an herbal tea (‘teng‐cha’), which has traditionally been used to alleviate respiratory infections, colds, coughs, sore throats and asthma. Dihydromyricetin (DHM), the most abundant (approximately 30%) flavonoid in *A. grossedentata*, possesses numerous bioactivities, including anti‐oxidative, anti‐inflammatory and anti‐cancer effects 9, 10, 11, 12, 13. Flavonoids, including quercetin, kaempferol, chrysin and luteolin, have been reported to improve insulin sensitivity without promoting adipogenesis 14, 15, 16. Our previous study revealed that DHM improved insulin sensitivity without causing excessive body weight gain *in vivo* 17. We hypothesized, therefore, that DHM might inhibit the MEK/ERK signalling pathway and thus down‐regulate phosphorylation of PPARγ at Ser273.

In this study, we investigated the effects of DHM on adipogenesis and glucose uptake and also explored the molecular mechanisms underlying the pharmacological effects of DHM using 3T3‐L1 adipocytes. We found that DHM attenuated adipogenesis and insulin resistance in adipocytes by inhibiting phosphorylation of PPARγ at Ser273 and that this was, at least in part, associated with modulation of the MEK/ERK signalling pathway.

## Materials and methods

### Chemicals and reagents

High glucose Dulbecco's Modified Eagle's Medium (DMEM) and foetal bovine serum (FBS) were purchased from Hyclone Laboratories, Inc. (Logan, UT, USA). Bovine calf serum was purchased from Gibco Life Technologies (Thermo Fisher, Carlsbad, CA, USA). DHM (SML0295), rosiglitazone (ROSI, R2408), GW9662 (M6191), PD98059 (P215), 3‐isobutyl‐1‐methylxanthine (IBMX, I7018), dexamethasone (DEX, D4902) and insulin (I5500) were obtained from Sigma‐Aldrich (St. Louis, MO, USA). 2‐[*N*‐(7‐nitrobenz‐2‐oxa‐1,3‐diazol‐4‐yl) amino]‐2‐deoxyglucose (2‐NBDG; N13195) was obtained from Invitrogen (Thermo Fisher, Carlsbad, CA, USA). Antibodies against PPARγ, phospho‐CDK5 (Ser159), CDK5, phospho‐ERK1/2 (Thr202/Tyr204), ERK and β‐actin were purchased from Santa Cruz Biotechnology, Inc. (Dallas, TX, USA). Antibody against fatty acid‐binding protein 4 (FABP4) was purchased from Cell Signaling Technology, Inc. (Beverly, MA, USA). Antibody against phospho‐PPARγ (Ser273) was obtained from Biosynthesis Biotechnology Co., Ltd. (Beijing, China).

### Cell culture and differentiation

Mouse embryonic fibroblast 3T3‐L1 cells were obtained from the Cell Bank of the Chinese Academy of Sciences (Shanghai, China). Cells were maintained in DMEM containing 10% bovine calf serum at 37°C in a 5% CO_2_ incubator. Adipocyte differentiation was induced as previously described 18. In brief, 2 days after confluence, the medium was changed to differentiation medium containing DMEM, 10% FBS, IBMX (0.5 mM), DEX (1 μM) and insulin (5 μg/ml) for 48 hrs. DHM (1, 3 or 10 μM) or ROSI (10 μM) was added to the medium at the beginning of differentiation, and the effect on adipogenesis was determined. The medium was then replaced with DMEM containing 10% FBS, and 5 μg/ml insulin and the cells were incubated for an additional 48 hrs. Subsequently, the cells were maintained in DMEM containing 10% FBS, with the medium replaced every other day for 4 days, until more than 80% of the pre‐adipocytes had differentiated into adipocytes. To induce insulin resistance, DEX (1 μM) was added to the medium for 16 hrs after adipocyte differentiation. A glucose uptake assay was used to confirm that insulin resistance had been established.

### Cell proliferation assay

Cell proliferation was measured using a CCK‐8 kit (Dojindo, Kumamoto, Japan), according to the manufacturer's protocol. Briefly, cells were seeded in 96‐well plates (5000 cells/well) and incubated at 37°C overnight. DHM (0–80 μM) or 0.1% DMSO as control was added to the medium and, after incubation for 48 hrs, CCK‐8 reagent (10 μL/well) was added to the plates, and the cells were incubated at 37°C for 2 hrs. Absorbance was measured at 450 nm using a SpectraMax M2 microplate luminometer (Molecular Devices, Sunnyvale, CA, USA).

### Oil Red O staining

Differentiated 3T3‐L1 adipocytes were washed with phosphate‐buffered saline (PBS), fixed with 10% formaldehyde for 1 hr and washed twice with PBS. The cells were then incubated with fresh Oil Red O working solution (0.5% in isopropanol diluted with water 3:2) for 30 min., washed with water and photographed using a CK40 inverted microscope (Olympus, Tokyo, Japan). The staining dye was dissolved in isopropanol and quantified using a microplate luminometer, with detection at 510 nm.

### Glucose uptake assay

The glucose uptake assay was performed using 2‐NBDG, as previously described 19. In brief, 3T3‐L1 adipocytes were seeded in 96‐well fluorescence plates and incubated at 37°C overnight. The culture medium was changed to serum‐free and low‐glucose DMEM for 1 hr. The cells were incubated with or without insulin (100 nM) for 20 min. and then incubated with 2‐NBDG (50 μM) at 37°C for 45 min. The cells were washed twice with ice‐cold PBS prior to fluorescence determination using a microplate luminometer (excitation wavelength of 485 nm and emission wavelength of 535 nm). To determine the effects of DHM on glucose uptake in insulin‐resistant adipocytes, differentiated 3T3‐L1 cells were pre‐incubated with DHM (1, 3 or 10 μM) or ROSI (10 μM) for 2 hrs and then treated with DEX (1 μM) for an additional 16 hrs. Glucose uptake was measured as described above.

### Adiponectin secretion assay

Fully differentiated 3T3‐L1 adipocytes were pre‐treated with DHM (1, 3 or 10 μM) for 2 hrs and then incubated with DEX (1 μM) for 16 hrs. The culture medium was collected, and the amount of adiponectin secreted by the adipocytes was determined using a mouse adiponectin enzyme‐linked immunosorbent assay (ELISA) kit (Enzyme‐linked Biotechnology Co., Ltd., Shanghai, China).

### Western blot analysis

Cells were lysed in radioimmune precipitation assay buffer (RIPA, Sigma‐Aldrich) containing 1% phenylmethanesulfonyl fluoride (PMSF, Sigma‐Aldrich). Cell suspensions were centrifuged at 15,000 r.p.m. for 15 min. at 4°C. Supernatants were collected, and proteins were quantified using a bicinchoninic acid assay. Equal amounts of protein were separated by sodium dodecyl sulphate polyacrylamide gel electrophoresis and transferred to polyvinylidene fluoride membranes. Membranes were blocked with 5% bovine serum albumin (BSA, Sigma‐Aldrich) and incubated with primary antibodies at 4°C overnight. After incubation with horseradish peroxidase secondary antibodies (Santa Cruz), protein bands were detected with enhanced chemiluminescent agents using a Fusion FX imaging system (Vilber Lourmat, Marne‐la‐Vallée, France) and quantified using Image‐Pro Plus version 6.0 image analysis software (Media Cybernetics, Rockville, MD, USA).

### Statistical analysis

Data are presented as mean ± S.E.M. Statistical analyses were performed by one‐way ANOVA, followed by Dunnett's *post hoc* test using GraphPad Prism 5.0 software (GraphPad Software, Inc., La Jolla, CA, USA). A *P* value <0.05 was considered to be statistically significant. All experiments were conducted in triplicate and repeated at least three times.

## Results

### Dihydromyricetin promotes glucose uptake and reduces adipogenesis in 3T3‐L1 adipocytes

**Figure 1:**
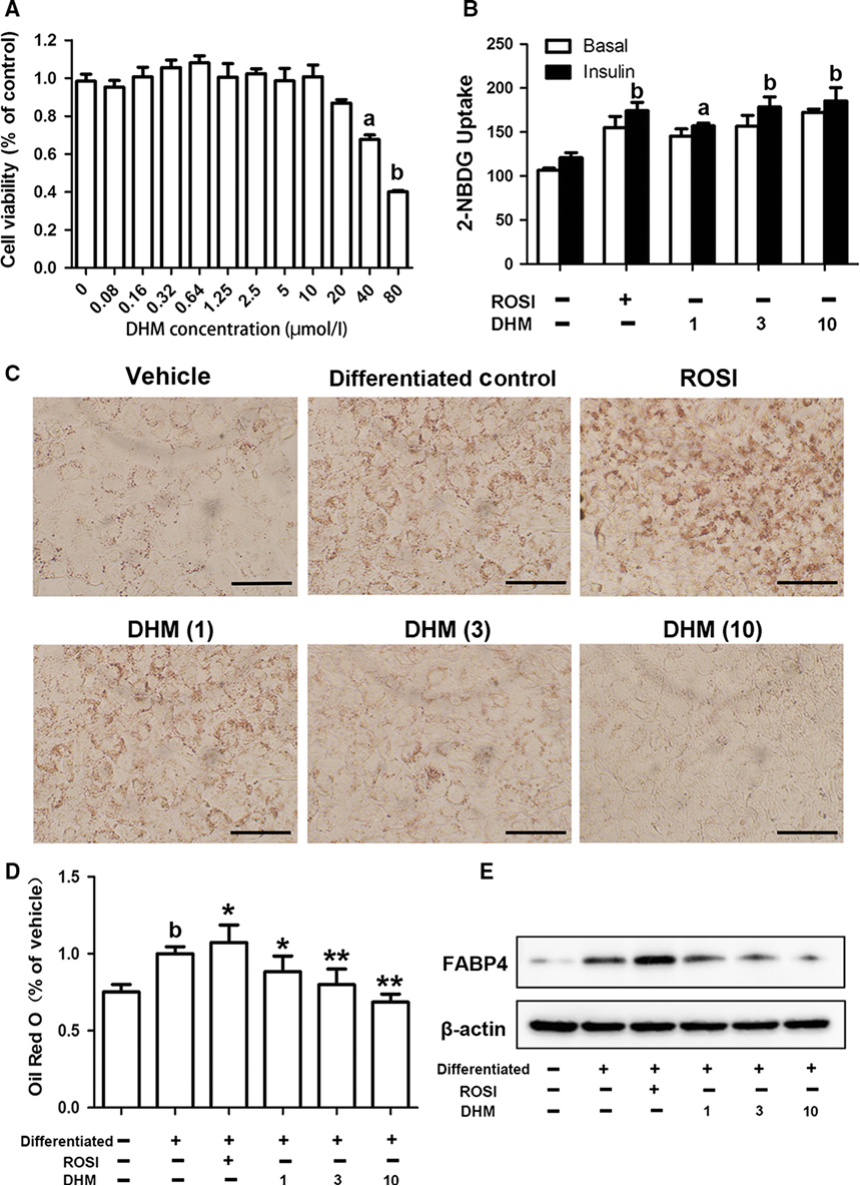
Effects of DHM on cell viability, adipogenesis and glucose uptake in 3T3‐L1 cells. 3T3‐L1 cells were treated with vehicle or differentiation medium for 8 days. ROSI (10 μM) or DHM (1, 3 or 10 μM) was added to the differentiation medium at the beginning of differentiation. **A** Cell viability was analysed using CCK‐8 kit. Data represent the mean ± S.E.M. of eight independent experiments. ^a^ *P* < 0.05, ^b^ *P* < 0.01 *versus* vehicle. **B** Glucose uptake was detected by 2‐NBDG uptake assay. Data are presented as the mean ± S.E.M. of five independent experiments. ^a^ *P* < 0.05, ^b^ *P* < 0.01 *versus* vehicle. **C** Lipid accumulation was visualized by Oil Red O staining (magnification: ×200). **D** Quantification of lipid drop dissolved in isopropanol with detection at 510 nm. Data are presented as the mean ± S.E.M. of five independent experiments. ^b^ *P* < 0.01 *versus* vehicle; *P < 0.05, **P < 0.01 *versus* differentiated control. **E** Protein expression of FABP4 determined by Western blot analysis.

DHM did not significantly suppress cell viability at concentrations lower than 40 μM (Fig. 1A) and dose dependently increased glucose uptake by 3T3‐L1 adipocytes (Fig. 1B). To determine the effect of DHM on adipogenic differentiation of 3T3‐L1 cells, cells were differentiated in the absence or presence of DHM (1, 3 or 10 μM). After 8 days differentiation, Oil Red O staining showed that DHM significantly decreased lipid accumulation, while ROSI (10 μM) increased adipogenesis of adipocytes (Fig. 1C). This was in line with quantitative analysis by isopropanol (Fig. 1D). DHM also markedly decreased expression of the adipogenesis marker FABP4 (Fig. 1E). Taken together, these findings suggest that DHM decreased adipogenic differentiation and improved glucose uptake by 3T3‐L1 adipocytes.

### Dihydromyricetin enhances glucose uptake and adiponectin secretion in DEX‐treated adipocytes

**Figure 2:**
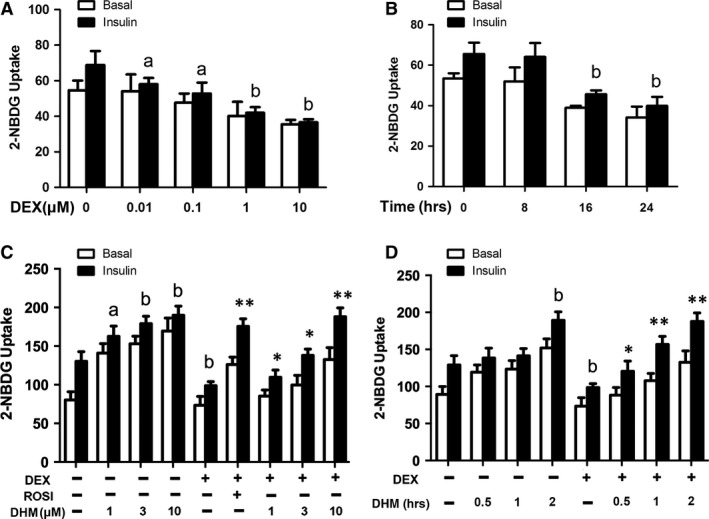
DHM improves glucose uptake and adiponectin secretion in DEX‐induced insulin‐resistant 3T3‐L1 cells. **A** Differentiated cells were incubated with DEX (0.01, 0.1, 1 or 10 μM) for 16 hrs and subsequently stimulated with or without insulin (100 nM) for 20 min. Glucose uptake was detected by 2‐NBDG uptake assay. **B** Differentiated cells were incubated with DEX (1 μM) for 8, 16 or 24 hrs and then stimulated with or without insulin (100 nM) for 20 min. Glucose uptake was detected by 2‐NBDG uptake assay. **C** Differentiated cells were incubated with DHM (1, 3 or 10 μM) for 18 hrs or pre‐treated with ROSI (10 μM) or DHM (1, 3 or 10 μM) for 2 hrs and subsequently incubated with DEX (1 μM) for another 16 hrs. **D** Differentiated adipocytes were incubated with DHM (3 μM) for different time intervals (0.5, 1 and 2 hrs) and treated with or without DEX (1 μM) for another 16 hrs. Glucose uptake was detected by 2‐NBDG uptake assay. Data are presented as the mean ± S.E.M. of six independent experiments. ^a^ *P* < 0.05, ^b^ *P* < 0.01 *versus* vehicle; *P < 0.05, **P < 0.01 *versus* DEX group.

DEX was used to induce insulin resistance in adipocytes as described previously. Differentiated adipocytes were incubated with DEX (0.01, 0.1, 1 or 10 μM) for 16 hrs or with DEX (1 μM) for different time intervals (8, 16 or 24 hrs). As shown in Figures 2A and B, DEX time and dose dependently decreased glucose uptake by 3T3‐L1 cells. Treatment with DEX (1 μM) for 16 hrs reduced glucose by 30.3% compared with vehicle (*P* < 0.05). To test the effect of DHM on insulin‐resistant adipocytes, differentiated 3T3‐L1 cells were pre‐treated with different indicated concentrations of DHM (1, 3, 10 μM) or different incubating time intervals (0.5, 1, 2 hrs). DHM significantly reversed the DEX‐induced decrease in glucose uptake (Figs 2C and D). DHM (10 μM) elevated glucose uptake by 90% compared with DEX group (*P* < 0.01).

### Dihydromyricetin enhances secretion of adiponectin and fibroblast growth factor 21 in DEX‐treated adipocytes

**Figure 3:**
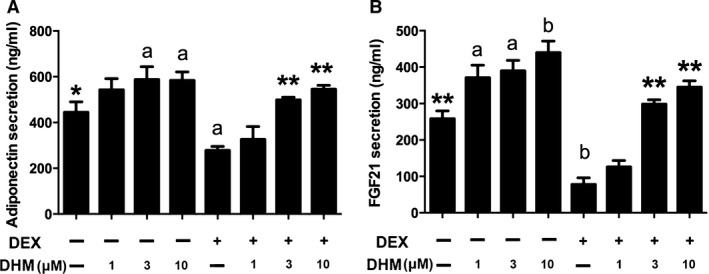
DHM increases adiponectin and FGF21 secretion in DEX‐treated 3T3‐L1 cells. Differentiated cells were incubated with DHM (1, 3 or 10 μM) for 18 hrs or pre‐treated with DHM (1, 3 or 10 μM) for 2 hrs and subsequently incubated with DEX (1 μM) for another 16 hrs. Culture media were collected, and levels of secreted adiponectin and FGF21 were determined using an ELISA kit. The secretion of adiponectin (**A**) and FGF21 (**B**) was determined by ELISA. Data are presented as the mean ± S.E.M. of five independent experiments. ^a^ *P* < 0.05, ^b^ *P* < 0.01 *versus* vehicle; *P < 0.05, **P < 0.01 *versus* DEX group.

Adiponectin is secreted predominantly by adipocytes and plays an important role in lipid and glucose metabolism. Another adipokine, fibroblast growth factor 21 (FGF21), also exerts metabolic effects, such as enhancing lipolysis and expression of adiponectin, through autocrine and/or paracrine signalling. As shown in Figure 3, DHM significantly increased secretion of adiponectin and FGF21 in adipocytes. Moreover, DHM dose dependently reversed the decrease of both adiponectin (*P* < 0.01, Fig. 3A) and FGF21 (*P* < 0.01, Fig. 3B) in DEX‐treated adipocytes, suggesting that the effects of DHM on secretion of adiponectin and FGF21 may contribute, at least in part, to increased glucose uptake by insulin‐resistant adipocytes.

### Dihydromyricetin down‐regulates ERK/CDK5 activity and inhibits phosphorylation of PPARγ at Ser273

**Figure 4:**
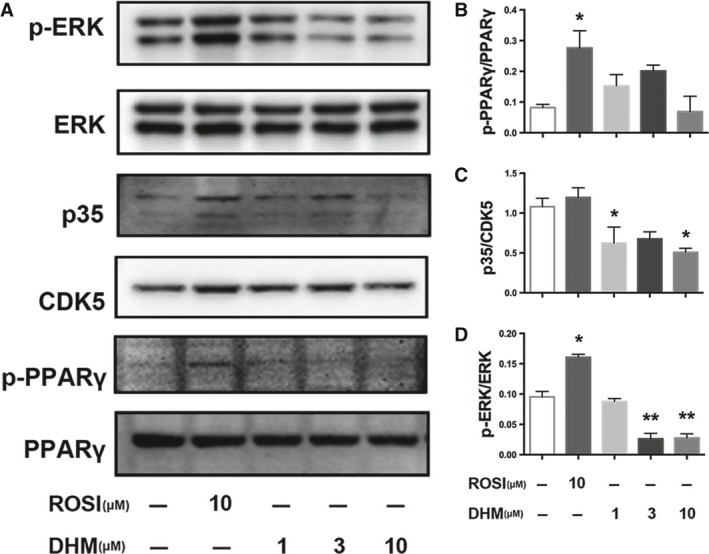
DHM inhibits phosphorylation of PPARγ at Ser273 and reduces ERK/CDK5 activity. Differentiated cells were incubated with ROSI (10 μM) or DHM (1, 3 or 10 μM) for 18 hrs. Protein expression was determined by Western blot analysis. **A** Bands of Western blot. **B** Densitometry analyses of p‐PPARγ/PPARγ, p35/CDK5 (**C**) and p‐ERK/ERK (**D**). Data are presented as the mean ± S.E.M. of four independent experiments. *P < 0.05, **P < 0.01 *versus* control.

**Figure 5:**
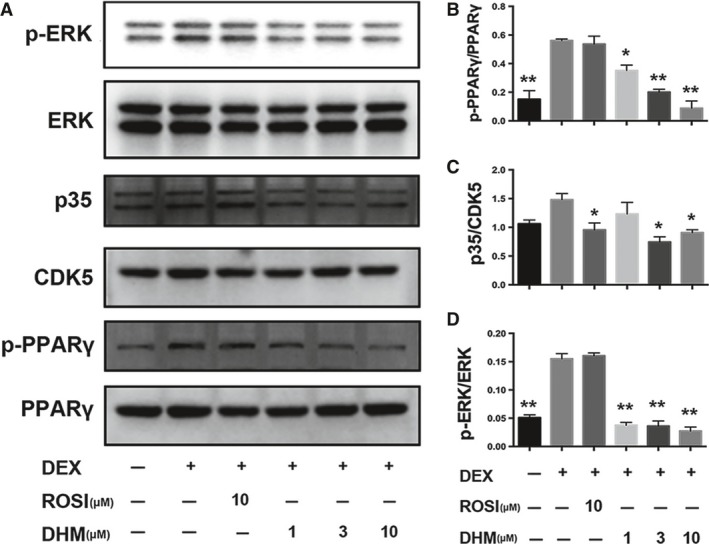
DHM inhibits phosphorylation of PPARγ at Ser273 and reduces ERK/CDK5 activity in DEX‐treated adipocytes. Differentiated cells were pretreated with ROSI (10 μM) or DHM (1, 3 or 10 μM) for 2 hrs and subsequently incubated with DEX (1 μM) for another 16 hrs. Protein expression was determined by western blot analysis. **A** Bands of western blot. **B** Densitometry analyses of p‐PPARγ/PPARγ, p35/CDK5 (**C**) and p‐ERK/ERK (**D**). Data are presented as the mean ± S.E.M. of four independent experiments. *p <0.05, **p <0.01 versus DEX group.

Nuclear receptor PPARγ plays a vital role in glucose and lipid metabolism. We previously showed that DHM improved insulin sensitivity, without causing excessive body weight gain, in Zucker diabetic fatty rats by inhibiting phosphorylation of PPARγ 17. We have now further investigated the possible role of DHM‐induced regulation of PPARγ phosphorylation in adipocytes. As shown in Figure 4A, in normal adipocytes, DHM did not significantly affect PPARγ phosphorylation (Fig. 4B) but markedly decreased CDK5 activation (*P* < 0.05, Fig. 4C) and ERK phosphorylation (*P* < 0.01, Fig. 4D). Notably, in DEX‐treated adipocytes (Fig. 5A), DHM dose dependently decreased phosphorylation of PPARγ at Ser273 (Fig. 5B). DHM reduced PPARγ phosphorylation more potently than ROSI. Interestingly, DHM showed different effects on CDK5 and ERK, the main mediators of PPARγ phosphorylation at Ser273. Low dose DHM (1 μM) had no significant effect on activation of CDK5, whereas higher doses (3 and 10 μM) decreased CDK5 activation, compared with differentiated control (Fig. 5C). DHM significantly decreased ERK phosphorylation, whereas ROSI significantly decreased CDK5 activation but had no effect on phosphorylation of ERK (Figs 5C and D).

### Inhibition of PPARγ reverses effects of dihydromyricetin on glucose uptake and adiponectin secretion in DEX‐treated adipocytes

**Figure 6:**
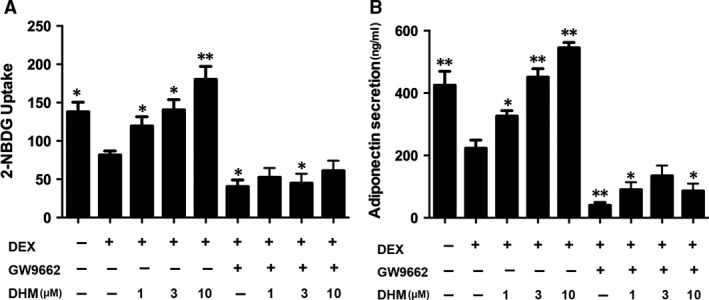
PPARγ inhibitor GW9662 blocks promotion of glucose uptake and adiponectin secretion by DHM in DEX‐treated adipocytes. Differentiated 3T3‐L1 adipocytes were pre‐treated with GW9662 (10 μM) for 1 hr and then co‐incubated with DHM (1, 3 or 10 μM) for 2 hrs before addition of DEX (1 μM) and incubation for another 16 hrs. **A** Glucose uptake was determined using a fluorescence microplate reader. **B** Adiponectin secretion was detected by ELISA. Data are presented as the mean ± S.E.M. of five independent experiments. *P < 0.05, **P < 0.01 *versus* DEX group.

To confirm that DHM promotes glucose and adiponectin secretion by regulating PPARγ, differentiated adipocytes were pre‐treated with PPARγ inhibitor, GW9662 (10 μM), for 1 hr before addition of DHM. GW9662 markedly decreased glucose uptake (*P* < 0.05, Fig. 6A) and adiponectin secretion (*P* < 0.01, Fig. 6B). GW9662 also reversed the beneficial effects of DHM in DEX‐treated adipocytes, suggesting that DHM promotes glucose uptake and adiponectin secretion in adipocytes by regulating PPARγ.

### Role of MEK inhibition on effects of dihydromyricetin in DEX‐treated adipocytes

**Figure 7:**
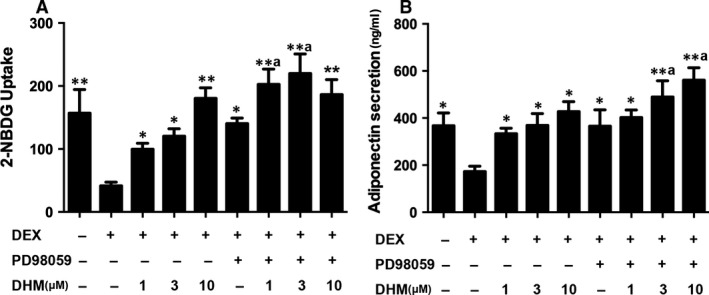
Effects of MEK inhibitor PD98059 on promotion of glucose uptake and adiponectin secretion by DHM in DEX‐treated adipocytes. Differentiated 3T3‐L1 adipocytes were pre‐treated with PD98059 (10 μM) for 1 hr and then co‐incubated with DHM (1, 3 or 10 μM) for 2 hrs before addition of DEX (1 μM) and incubation for another 16 hrs. **A** Glucose uptake was determined using a fluorescence microplate reader. **B** Adiponectin secretion was detected by ELISA. Data are presented as the mean ± S.E.M. of five independent experiments. ^a^ *P* < 0.05 *versus* DEX+PD98059 group; *P < 0.05, **P < 0.01 *versus* DEX group.

MEK phosphorylates and activates ERK, which is the kinase that phosphorylates PPARγ at Ser273. As DHM decreases phosphorylation of ERK, we compared the effects of DHM with those of MEK inhibitor PD98059. As shown in Figure 7, PD98059 (10 μM) significantly enhanced glucose uptake and adiponectin secretion in insulin‐resistant adipocytes (*P* < 0.05). Intriguingly, compared with PD98059 alone, DHM together with PD98059 acted synergistically and robustly improved glucose uptake and adiponectin secretion in adipocytes (*P* < 0.05). These results suggest that DHM inhibits MEK and thereby suppresses phosphorylation of ERK, resulting in inhibition of phosphorylation of PPARγ at Ser273.

## Discussion

Nuclear receptor PPARγ is predominantly expressed in adipose tissue and plays a crucial role in adipocyte differentiation, insulin sensitivity and energy homeostasis 20. PPARγ has, therefore, long been considered an attractive drug target for the treatment of diabetes. Although full PPARγ agonists, such as TZDs, improve insulin sensitivity and reduce blood glucose levels 21, 22, their clinical utility is restricted by side‐effects, including weight gain, fluid retention and increased risk of congestive heart failure 23, 24, 25. Recent studies have shown that inhibition of phosphorylation of PPARγ at Ser273 may provide a new approach to anti‐diabetic treatment 6, 7. Our previous study revealed that DHM improved insulin sensitivity *in vivo*, without causing excessive body weight gain, by inhibition of PPARγ phosphorylation 17. In the present study, we have demonstrated the effects of DHM on differentiation and glucose uptake in adipocytes. We found that DHM enhanced adiponectin secretion in DEX‐treated adipocytes and also reduced the activity of MEK/ERK, thus inhibiting phosphorylation of PPARγ at Ser273. Together, these results support a novel role for DHM in improving insulin sensitivity without causing fat accumulation in 3T3‐L1 adipocytes, probably through modulation of the MEK/ERK signalling pathway.

Adipocytes play vital roles in regulating energy balance and glucose homeostasis. Cross‐sectional and prospective studies indicate that enlarged adipocyte size is associated with insulin resistance 26. Hypertrophic adipocytes secrete more proinflammatory factors, such as TNF‐α and IL‐6, which impair insulin‐stimulated glucose uptake through effects on glucose transporter type 4 (GLUT‐4), insulin receptor autophosphorylation and insulin receptor substrate‐1 27. Many flavonoids, including epigallocatechin gallate (EGCG) and myricetin, have been reported to increase glucose uptake in adipocytes. In this study, DHM was shown to significantly decrease differentiation of pre‐adipocytes and to reverse DEX‐induced reduction of glucose uptake in 3T3‐L1 adipocytes. Although, 3T3‐L1 cells are widely used to explore pathogenesis of diabetes *in vitro*, there still are discrepant results between this cultured cell line and primary adipocytes. Glucose uptake is stimulated 20‐ to 30‐fold by insulin in rat adipocytes, but only threefold to fivefold in 3T3‐L1 adipocytes. This is probably due to more abundant expressed insulin‐insensitive transporter GLUT1 in 3T3‐L1 cells, which enhance basal (non‐insulin stimulated) glucose uptake 28. Furthermore, the cultured cells have a reduced capacity to sequester insulin‐sensitive transporter GLUT4 intracellularly in the basal (unstimulated) state and, consequently, a blunted insulin response 29. Therefore, in this study, insulin‐stimulated glucose uptake is not significantly enhanced compared with basal state. Notably, DHM down‐regulated FABP4, which is predominantly located in adipocytes and is the downstream target gene modulated by PPARγ 30. FABP4 regulates metabolic and inflammatory pathways, and its down‐regulation can alleviate insulin resistance and atherosclerosis 31.

Adipose tissue accounts for only 10–15% of glucose disposal after a meal, which is much less than muscle 32. The fact that PPARγ ligands, such as TZDs, have excellent anti‐diabetic activity suggests that adipose tissue regulates organismal glucose homeostasis through mechanisms other than glucose disposal. In the last two decades, the endocrine function of adipose tissue has attracted great attention because of its role in maintaining glucose homeostasis. Adiponectin, one of adipokines secreted into the circulation by adipocytes, enhances glucose transport and improves insulin sensitivity in muscle and liver by activating AMP‐activated kinase (AMPK) 33. In this study, DHM significantly increased adiponectin secretion in DEX‐treated adipocytes. Many flavonoids, such as tangeretin, catechin and cyanidin 3‐glucoside, have been shown to increase adiponectin expression and secretion, both *in vivo* and *in vitro* 34, 35, 36, 37. Both our previous study, which showed that DHM increased circulating levels of adiponectin *in vivo*, and our present results are in agreement with these other studies. Another adipokine, FGF21, exerts metabolic effects in adipose tissue in an autocrine and/or paracrine manner. FGF21 not only activates thermogenesis in brown and beige adipocytes but also enhances the expression and secretion of adiponectin in white fat tissue 38, 39. In the present study, DHM markedly enhanced FGF21 secretion in adipocytes. Together with the elevation of adiponectin levels, these results suggest that DHM may improve insulin sensitivity through the FGF21‐adiponectin axis.

Once activated by ligand binding, PPARγ binds to DNA and modulates not only adipogenesis genes such as lipoprotein lipase, *Fabp4*, phosphoenolpyruvate carboxykinase and acyl‐CoA synthetase 40, but also genes regulating glucose metabolism, such as GLUT4 and adiponectin 41. Accumulating evidence suggests that modulating, rather than fully activating, PPARγ might improve glucose metabolism whilst preventing excessive adipogenesis 42. Aside from classical receptor transcriptional agonism, post‐translational regulation of PPARγ was associated with diabetogenic gene expression in adipose tissues. Inhibition of CDK5‐induced phosphorylation of PPARγ at Ser273 is a distinct mechanism for anti‐diabetic drugs targeting PPARγ 6. Partial agonist or non‐agonist PPARγ ligands exert potent anti‐diabetic effects by blocking phosphorylation of PPARγ at Ser273, without promoting adipogenesis 7, 8. In this study, DHM showed no effect on total PPARγ protein levels but significantly decreased phosphorylation of PPARγ at Ser273. The effects of DHM could be blocked by GW9662, a potent and irreversible antagonist of PPARγ, suggesting that DHM, at least partially, enhances glucose uptake and adiponectin secretion *via* PPARγ‐specific pathway. However, GW9662 might exert non‐PPARγ‐specific effects as well. Besides PPARγ antagonism, GW9662 inhibits adipocytes differentiation through regulating connective tissue growth factor (CTGF) mRNA expression 43, which inhibits the adipocyte differentiation *via* the C/EBP pathway 44. Hence, we cannot rule out the possibility of other non‐PPARγ‐specific pathways underlying the protective effects of DHM.

Recently, another ERK kinase was found to directly phosphorylate PPARγ at Ser273 8. The mitogen‐activated protein kinase (MAPK) pathway is involved in maintaining energy homeostasis. As well as phosphorylating PPARγ at Ser273, leading to insulin resistance, the MEK/ERK pathway is a major regulator of energy expenditure and inflammation. Activation of ERK by overexpression of MEK causes decreased energy expenditure and systemic insulin resistance 45. The secretion of proinflammatory cytokines, including TNF‐α and IL‐6, is strongly elevated both in chronic inflammatory and insulin‐resistant states in adipose tissue macrophages. Inhibition of the MEK/ERK pathway, however, markedly ameliorated insulin resistance by decreasing IL‐6 production in monocyte 46. Administration of MEK inhibitors improved dysregulation of adiponectin secretion and enhanced insulin sensitivity in *db/db* mice and KKAy diabetic mice fed a high fat diet 47. Previous studies have shown that flavonoids, including luteolin, quercetin and kaempferol, exert protective effects against cancer and acute lung injury by inhibiting the MEK/ERK pathway 48, 49, 50. Consistent with these findings, we found that flavonoid DHM significantly down‐regulated ERK phosphorylation in DEX‐treated adipocytes, suggesting that DHM could block phosphorylation of PPARγ at Ser273 *via* inhibiting the MEK/ERK pathway. However, more efforts are needed to elucidate the underlying mechanisms, particularly the interactions between DHM and ERK. Taken together, the results show that inhibition of the MEK/ERK pathway improves insulin resistance not only by down‐regulating PPARγ phosphorylation, but also through PPARγ‐independent reduction of inflammatory cytokine secretion by adipocytes and monocytes. Intriguing, DHM acted synergistically with the MEK inhibitor PD98059 to enhance glucose uptake and adiponectin secretion in adipocytes, suggesting a novel strategy of combination DHM with MEK inhibitor to treat type 2 diabetes.

In summary, our study has further elucidated the molecular mechanism underlying the anti‐diabetic effect of DHM. For the first time, we propose that DHM inhibits phosphorylation of PPARγ at Ser273 by inhibition of the MEK/ERK pathway. Although more work is needed to fully characterize the intermolecular interactions, our results suggest that DHM is a promising treatment for type 2 diabetes. Our work lays a foundation for future research to explore the preventive and therapeutic utilization of natural phytochemicals, such as DHM, in type 2 diabetes.

## Conflict of interest

The authors declare that there are no conflicts of interest.
